# Sex Differences in Spiritual Coping, Forgiveness, and Gratitude Before and After a Basic Alcohol Addiction Treatment Program

**DOI:** 10.1007/s10943-015-0002-0

**Published:** 2015-01-20

**Authors:** Edyta Charzyńska

**Affiliations:** Department of Psychology, University of Social Sciences and Humanities, Chodakowska 19/31, 03-815 Warsaw, Poland

**Keywords:** Spirituality, Alcohol dependence, Spiritual coping, Forgiveness, Gratitude

## Abstract

The aim of the study was to examine the sex differences in the initial level of spiritual coping, forgiveness, and gratitude and changes occurring in these areas during a basic alcohol addiction treatment program. The study involved 112 persons, including 56 women and 56 men, who started and completed a basic alcohol addiction treatment program at day care units of 11 treatment centers. Two measurements were taken: one in the first week of the treatment, and one in the last week (5th–7th week after baseline). The Spiritual Coping Questionnaire, the Forgiveness Scale, and Gratitude Questionnaire were used. When starting the therapy, women had a higher level of negative spiritual coping (*p* = .024) and a lower level of forgiveness of others (*p* = .041) than men. During the therapy, positive changes in spiritual coping occurred in both sex groups, although in the case of women they involved improvements in more domains and they were stronger. The increase in the level of moral values (except for self-forgiveness) was noted solely in women. The study reveals the need to take sex differences into consideration when introducing spiritual elements into the therapy.

## Introduction

It was only in the 1970s and 1980s that researchers and practitioners noticed how little was known on the problem of alcoholism among women (Smith and Weisner 2000). Before, a belief had been prevalent that addiction of a woman is not different from that of a man. In the following years, there was a breakthrough: the specificity of women’s alcoholism was gradually noticed, which resulted in the development of research devoted to sex differences in the addiction etiopathogenesis, the consequences of drinking for biopsychosocial health, barriers in staring therapy, factors promoting relapses, and the results of treatment (Green 2006).

Although currently sex differences regarding alcohol problems are not as large as in the past, women still drink alcohol less often, consume it in smaller amounts, and get addicted to it less frequently (Greenfield et al. 2003; Wilsnack et al. 2009). On the other hand, although they start drinking at a later age, once they are addicted, they more quickly lose control of drinking than men do, they have greater problems connected with drinking, and they suffer greater health consequences (Randall et al. 1999).

### Spiritual Sphere and Alcoholism

Although the religiousness and spirituality terminology is still quite unclear, many agree that religiousness and spirituality are not the same concept, and despite sharing some areas, they may differ (Emmons 1999; Paloutzian and Park 2005). It is sometimes assumed that religiousness is rather institutional, external, related to religious principles, and a specific rite, whereas spirituality has a more internal and personal character. Spirituality may be manifested by looking for one’s personal authenticity, completeness, the meaning and purpose of life, the will to help others for the benefit of humanity, and the development of bond with the Higher Being or another force beyond human existence and knowledge (Love and Talbot 1999).

The relationships between religiousness or spirituality and alcoholism are one of the important themes in the contemporary psychology of addictive behaviors. They are treated as factors that protect one from various hazardous behaviors, including addictions (Bliss 2007). Some researchers and therapists interpret the development of addiction as a consequence of inability to find the meaning of one’s life, the feeling of spiritual emptiness (Warfield and Goldstein 1996). On the other hand, alcohol addiction is often connected with the destruction of the spiritual sphere. The person starts to make up for low self-esteem, lack of satisfaction with relationships, and acute sense of purposelessness of life with alcohol, which becomes the center of life, replacing real values.

Many studies have confirmed that positive changes in the spiritual sphere of functioning occur during the addiction treatment. For example, in a longitudinal study conducted among 364 persons addicted to alcohol, Robinson et al. (2011a) observed an increase in private spiritual and religious practices, everyday spiritual experiences, and the sense of purposefulness of life after 6 months from baseline. In another study, spiritual growth of people participating in a 4-week addiction treatment was noted (Sterling et al. 2007). Additionally, this study proved that people who maintained their abstinence after 3 months of the beginning of treatment continued growing spiritually, whereas those who relapsed experienced a decrease in spirituality level. Research results also confirm that introducing issues connected with spirituality into the therapy, as well as attending AA meetings, lead to a higher probability of maintaining sobriety and improvement of life quality (Bliss 2007; Wnuk 2007; Robinson et al. 2011b).

### Spiritual Coping with Stress

In the face of a stressful situation, humans use various resources available to them. People who have a problem with alcohol are usually those who have not developed constructive methods of coping with difficult life situations (Mroziak et al. 1999). It is also known that the use of adaptive methods of coping increases the probability of completing the therapy and maintaining abstinence, while avoidance strategies and those that involve giving up any activity lower the chance of positive therapy results (Chodkiewicz 2005).

One source of constructive methods of coping may be the spiritual sphere. A lot is written about religious coping in the literature on the subject (Pargament 1997). A human may refer to God to look for support and hope in the difficult situation what usually has a positive influence on their functioning (Gall 2000; Zwingmann et al. 2006). Nevertheless, it should be remembered that religious coping is not always positive and beneficial for an individual. Pargament et al. (2003) also identify negative religious ways of coping. One may, e.g., blame the Higher Being for their failures, divert from it or negate its existence what usually has a deleterious impact on their health (Zwingmann et al. 2006).

Establishing or maintaining the relation with God, seeking support, and love in Him are not the only ways of using the spiritual potential to combat a stressful situation. One can also concentrate on their internal life, deepen self-awareness, and establish valuable relationships with other people or a close contact with Nature. Hence, just like spirituality is distinguished from religiousness, spiritual coping—understood as attempts to overcome the stressor on the basis of one’s non-material resources—can be differentiated from religious coping (Baldacchino and Draper 2001; Charzyńska 2014). Research on spiritual coping is still in its preliminary phase. Previous works on the conceptualization and operationalization of this term have allowed to distinguish the following aspects and domains of spiritual coping (Charzyńska 2014):

- positive spiritual coping in the personal domain—pursuit of a goal, sense, and meaning; concentration on one’s internal life; acquiring more and more self-knowledge; looking for internal peace and harmony,
- positive spiritual coping in the social domain—establishing and maintaining deep and valuable relations with other people, heeding moral values, caring about others, willingness to help, displaying love, empathy, and compassion,
- positive spiritual coping in the environmental domain—seeking closeness to Nature, perceiving harmony in it, and treating Nature as friendly to humans,
- positive spiritual coping in the religious domain—maintaining stable relation with God/Higher Being, based on the sense of presence, love, and trust,
- negative spiritual coping in the personal domain—negating the goal and meaning of one’s life, emphasizing one’s weaknesses, concentration on one’s transgressions,
- negative spiritual coping in the social domain—perceiving people as egoistic, which results in aversion, hostility, or envy toward others,
- negative spiritual coping in the religious domain—holding a grudge toward God/Higher Being, blaming Him/It for one’s own failures, negating His/Its love, and care for humans.

The methods of spiritual coping described above may be activated when confronting stress accompanying an illness, also in the case of people addicted to alcohol, regardless of whether these people believe in God or not.

### Forgiveness

Forgiveness is one of the most important moral values in many religious traditions and spiritual formations (Jankelevitch 1967). As far as psychology is concerned, interest in forgiveness has recently occurred in positive psychology (Snyder and McCullough 2000). Peterson and Seligman (2004) included forgiveness in the group of 24 character strengths, indicating its important role in one’s functioning. For the development of research concerning forgiveness, it was crucial to recognize the fact that forgiveness does not only refer to religious or spiritually minded people but is rather universal and can characterize each person or group (Webb 2003). Up to date, many studies have confirmed the relationships between forgiveness and physical and mental health (Toussaint and Webb 2005; Witvliet et al. 2001). Forgiveness occurs when a harmed person is able to reduce their negative emotions, thoughts, and behaviors toward the transgressor (Gassin and Enright 1995). Some of the definitions also include the importance of arousing positive emotions toward the perpetrator in oneself (such as compassion, generosity, or even love; Enright and The Human Development Study Group 1991). It is also emphasized that this is an internal process chosen by the victim, and that it should not be mistaken for phenomena such as condoning, pardoning, denying, forgetting, excusing, or reconciling (Enright and Coyle 1998).

Initially, forgiveness was treated as a unidimensional construct, limited to forgiveness of others. Currently, its many aspects are extracted—different methods (e.g., offering, seeking, and feeling) and targets (self, others, deity, community; Toussaint and Webb 2005) of forgiveness are identified. Apart from forgiveness of others, considerable attention is given to self-forgiveness. It is defined as freeing oneself from negative emotions (such as shame or guilt) caused by committing a deed considered by the individual as morally reprehensible and replacing these emotions with beliefs, feelings, and behaviors beneficial for oneself (Hall and Fincham 2008). Another aspect of forgiveness, feeling forgiven by God, involves the recognition that one’s own offenses and acts considered by the person as morally wrong have been forgiven by God/the Higher Being (Toussaint et al. 2001).

Developing the ability to forgive seems to have a special role in the treatment for people addicted to alcohol (Worthington et al. 2006). On the one hand, a person who abuses alcohol charges others with the responsibility for their addiction, and on the other hand, in the periods of abstinence, he or she can suffer from overwhelming feelings of shame and worthlessness, which often results in discontinuance of abstinence (Wiechelt 2007). People who strongly believe in God may additionally feel it is impossible to reconstruct the relationship with the Higher Being because of their transgressions and diverting from God. If it turns out during the implementation of the 12 steps that negative emotions caused by the lack of ability to forgive are responsible for continuous alcohol abuse, then the stress is put on developing the ability to forgive oneself and others, mediated by the unlimited love of the forgiving God (Alcoholics Anonymous 1972). There are only few studies available that explore forgiveness in alcohol addiction and during the treatment. One of the study areas is changes in forgiveness occurring during the therapy. A research conducted by Sterling et al. (2007) showed that forgiveness significantly increased over a 4-week addiction treatment. In a study by Robinson et al. (2011a), in which forgiving and its different aspects were measured twice (at the beginning of the therapy and 6 months later), a slight increase in forgiving others and the general forgiveness level was observed. There were no significant changes in the levels of forgiving oneself and the feeling of being forgiven by God. Studies exploring the relationships of forgiveness with physical and mental functioning in addicted people show a positive effect of forgiveness on health (Webb et al. 2009), as well as treatment success (Webb et al. 2006). It is worth noting that research has proved some differences in the impact of forgiveness aspects on alcohol-related outcomes, confirming consistently the most beneficial role of self-forgiveness (Webb et al. 2006, 2009; Robinson et al. 2011b).

### Gratitude

Just like forgiveness, gratitude is also one of the key elements of world’s religions, providing the basis for ethics (Peterson and Seligman 2004). Psychologists took interest in gratitude in the 1990s. One of the more important reasons for paying attention to this moral virtue was the discovery of its relation with health (McCullough et al. 2002; Froh et al. 2009), and with pro-social behaviors (Bartlett and DeSteno 2006).

Gratitude is defined in different ways, e.g., it is treated as a moral value, attitude, emotion, habit, feature of personality, or a response to a stressful situation (Emmons and McCullough 2003). According to Emmons (2004, p. 9), it means the recognition of and appreciation for an altruistic gift. It involves three components such as: (1) a warm sense of appreciation for somebody or something, (2) a sense of goodwill toward that person or thing, and (3) a disposition to act that flows from appreciation and goodwill (Fitzgerald 1998).

Gratitude is treated as an important subject during the meetings of Alcoholics Anonymous (1972). When consuming alcohol, an individual concentrates on themselves, cannot appreciate the good received every day from God and other people, and cannot express gratitude for themselves. Maintaining sobriety should lead to noticing the gifts one receives, thanks to which their feelings of grief, anger, or loneliness can be replaced with positive emotions, such as gratitude. Although the topic of gratitude is mentioned at AA meetings, it is much more rarely discussed during the therapy. So far, it has not been the subject of studies of alcohol addiction either, although this moral value might be significant for maintaining abstinence and then the process of sobering.

### Aim of the Study and Hypotheses

The aim of the presented study was to examine the sex differences in spiritual coping, forgiveness, and gratitude before and after a basic alcohol addiction treatment program. So far, the studies in this area have not involved either multidimensional strategies of spiritual coping or gratitude. What is even more important, the differences between sexes regarding changes that occur in spiritual aspects during the therapy have not yet been studied. Taking into consideration the fact that most of the addicted are men, combining men and women in studies of spirituality may have caused the detection of patterns that are mostly characteristics of men, without considering the specificity of changes among women.

The following main hypotheses were made in this study:

#### Hypothesis 1

There will be a difference between women and men entering and completing a basic alcohol addiction treatment program in the level of spiritual coping, forgiveness, and gratitude.

On the basis of the current literature, it was impossible to predict the direction of this difference, which is why the indirectional hypothesis was formulated.

#### Hypothesis 2

During the therapy, both in women and in men, the use of positive spiritual strategies of coping with stress increases, the use of negative spiritual strategies decreases, and the levels of forgiveness and gratitude increase.

This hypothesis was made on the basis of the results of studies indicating positive changes in spiritual functioning which occur during an alcohol addiction therapy (Robinson et al. 2011a; Sterling et al. 2007).

#### Hypothesis 3

Changes in the elements of spiritual life that occur during the therapy are stronger and more numerous in women than in men.

It was considered as plausible that the general higher level of religiousness, spirituality, and moral values in women in comparison with men (Bryant 2007; Miller et al. 2008; Kashdan et al. 2009), as well as women’s higher tendency to use spiritual resources in a stressful situation (Strawbridge et al. 2000; Koenig et al. 1999), would result in a higher rate of positive changes in spiritual coping and moral values during the treatment.

## Methods

### Participants

Data being part of a greater database were used in the study. The total of 343 persons addicted to alcohol (including 98 women and 245 men) undergoing a short-term (6–8 weeks depending on the treatment center) group addiction treatment program took part in the study.

The basic treatment program was completed by 181 persons, including 125 men (51.02 %) and 56 women (57.14 %). Only data from persons who completed the therapy were used in the study: 56 men were coupled with 56 women, considering the age and education criteria.

The men’s mean age was *M* = 41.89 years, SD = 10.38, and the women’s was *M* = 42.39 years, SD = 10.91 (*t* (110) = .25; *p* > .05). The average addiction period in the case of men was *M* = 15.25 years, SD = 9.72, and of women *M* = 11.83 years, SD = 8.34 (*t* (104) = 1.94; *p* = .055). The other characteristics of the studied sample divided into sexes are presented in Table 1. There was a significant difference between men and women regarding the professional status (*χ* ^2^ (2) = 11.833; *p* = .003).

**Table 1:** Characteristics of the sample

Variables	Men (*n* = 56)	%	Women (*n* = 56)	%	*χ* ^2^	*p*
Education						
Elementary or lower secondary	9	16.1	9	16.1	0	ns
Vocational	21	37.5	21	37.5		
Secondary	20	35.7	20	35.7		
Higher	6	10.7	6	10.7		
Marital status						
Single	13	23.2	10	17.8	1.98	ns
(in)Formal relationship	29	51.8	29	51.8		
Divorced or separated	9	16.1	13	23.2		
Widow/widower	5	8.9	3	5.4		
N/A	0	0	1	1.8		
Professional status						
Employed	29	51.8	14	25.0	11.83	.003
Unemployed	18	32.1	36	64.3		
Pensioner	9	16.1	6	16.1		
Level of faith						
Deeply religious	5	8.9	5	8.9	.78	ns
Religious	33	58.9	36	64.3		
Undecided	9	16.1	6	10.7		
Irreligious	9	16.1	8	14.3		
N/A	0	0	1	1.8		
Court order for treatment						
Yes	8	14.3	4	7.1	1.49	ns
No	48	85.7	52	92.9		
Other addictions						
Yes	22	39.3	21	37.5	.04	ns
No	34	60.7	35	62.5		
Somatic disease						
Yes	18	32.1	14	25.0	.70	ns
No	38	67.9	42	75.0		
Mental illness (except addiction)						
Yes	16	28.6	18	32.1	.17	ns
No	40	71.4	38	67.9		
Attendance at AA meetings						
Yes	43	76.8	39	69.6	.7	ns
No	12	21.4	16	28.6		
N/A	1	1.8	1	1.8		

### Tools

#### Spiritual Coping

The Spiritual Coping Questionnaire (Charzyńska 2014) was used to measure spiritual coping with stress. The tool includes 32 items divided into seven subscales, constituting two main scales: positive and negative spiritual coping. Positive spiritual coping is made up of four subscale domains: personal (four items; item example: *I was trying to find inner peace within myself*), social (six items, e.g., *I was nurturing my attitude of love toward other people*), environmental (five items, e.g., *I was seeking closeness to Nature*), and religious (six items, e.g., *I was turning to God/Higher Being with every matter important for me*). Negative spiritual coping consists of three subscale domains: personal (four items, e.g., *I was convincing myself that my life had no goal whatsoever*), social (four items, e.g., *I was trying to prove to other people that they were egoists*), and religious (three items, e.g., *I was accusing God/Higher Being of what happened in my life*).

In the current study, questionnaire items were preceded by the following instruction: “The statements presented below refer to different ways of coping with difficult life events. Please indicate how well each of the statements describes what you did in the past 4 weeks when dealing with your drinking problem.”

The answers are given in a 5-point Likert scale: 1—“very inaccurately,” 2—“rather inaccurately,” 3—“neither inaccurately nor accurately,” 4—“rather accurately,” and 5—“very accurately.” The general results are calculated by averaging the results obtained in each subscale of spiritual coping. The results of particular subscales are obtained by averaging the responses to the appropriate items of the questionnaire.

The questionnaire’s internal consistency measured with the Cronbach’s *α* is satisfactory, and for the positive spiritual coping scale, it is .92; for negative spiritual coping, it is .82, and for individual subscales, it ranges from .71 to .94. Absolute stability measured with the test–retest method is also satisfactory. The construct and criterion validity of the tool are confirmed (Charzyńska 2014).

#### Forgiveness

Forgiveness was measured with a Polish adaptation (Charzyńska and Heszen 2013) of indices of forgiveness proposed by Toussaint et al. (2001). The Polish version of the tool, called the Forgiveness Scale, includes three aspects of forgiveness: forgiving oneself (two items), forgiving others (five items), and the feeling forgiven by God (two items). These subscales are the part of a high-order factor called “forgiveness.”

The questionnaire’s items are evaluated in a 5-point Likert scale with the use of two formats of response categories (depending on the item): “strongly agree,” “agree,” “hard to say,” “disagree,” “strongly disagree,” or “never,” “hardly ever,” “not very often,” “quite often,” “very often.” In the Polish version, the response categories were modified by including the option “not applicable,” for questions concerning one’s religiousness.

Some items in the questionnaire refer to the inclination to forgive, while others express the opposite tendency, so certain items need to be recoded. The results of subscales are calculated as the mean of the items they include, whereas the general level of forgiveness is estimated by averaging the results obtained in each subscale of forgiveness.

The reliability of the Forgiveness Scale measured with Cronbach’s *α* was, respectively, .75 for the general index of forgiveness, .65 for the “self-forgiveness” subscale, .74 for the “forgiveness of others” subscale, and .91 for the “feeling forgiven by God” subscale (Charzyńska and Heszen 2013). The absolute stability was also satisfactory. The construct validity of the tool was confirmed too, by means of a confirmatory analysis and relations of forgiveness with spirituality, religious coping, and gratitude. The criterion validity of the tool was confirmed by its correlation with the indices of physical and mental health (Toussaint et al. 2001; Charzyńska and Heszen 2013).

#### Gratitude

The level of gratitude was measured with the use of the Polish adaptation of a popular tool, Gratitude Questionnaire (GQ-6; McCullough et al. 2002), developed by Kossakowska and Kwiatek (2012).

The Gratitude Questionnaire comprises six items evaluated on a 7-point Likert scale, where 1 means “strongly disagree,” and 7—“strongly agree.” The scale is homogeneous: it involves 1 factor, called “gratitude” (Kossakowska and Kwiatek 2012). Four items refer to gratitude, and two to difficulty with expressing or feeling gratitude, so they have to be recoded at the calculation of results. The level of gratitude is obtained by adding up all the items.

The reliability of the GQ-6 scale calculated with Cronbach’s *α* coefficient was .72. The analysis of construct validity confirmed the relationships between gratitude and personality traits (the “Big Five,” especially agreeableness and extraversion), spirituality, life satisfaction, and forgiveness without taking revenge (Kossakowska and Kwiatek 2012).

### Procedure

The study was conducted between May and December 2012 at day care wards of 11 alcohol addiction treatment centers in Poland. These centers implement therapeutic elements originating in different traditions, although the strategic–structural approach dominates (Mellibruda 1997), which refers to the Minnesota model with elements of cognitive-behavioral and interactive approach. The Minnesota model, also called abstinence model, emphasizes the fact that alcoholism is an involuntary, primary disease, and has a chronic and progressive nature (Anderson 1981). It approaches addiction from many perspectives, taking into consideration the physical, psychological, social, and spiritual aspects. The therapy is mostly based on the philosophy and experiences of the Alcoholics Anonymous movement. The strategic–structural model (Mellibruda 1997) also emphasizes factors that increase the risk of addiction, including an orientation manifested in self-destructive tendencies, destructive patterns of interpersonal relations, and the degradation of one’s system of values. Although the role of spirituality is not as important as in the Minnesota model, the subject of devastation of spiritual life as a result of drinking is mentioned during the therapeutic sessions, especially the degradation of interpersonal relations, including moral values, and the need of restoring one’s spiritual life so as to keep abstinence and continue the process of sobering is stressed. Attending the meetings of Alcoholics Anonymous and implementing the 12-step program is also recommended.

The measurement was taken twice: in the first week of the basic therapy and in the last week of therapy (5th–7th week after baseline, depending on the center). The study was carried out by instructed fourth and fifth year’s psychology students. In two of the centers, the measurements were taken by day care ward therapists. The study was performed in groups of 3–6 persons. All the participants had signed informed consent forms, explaining the objective and course of the study, and emphasizing the voluntary and anonymous nature of participation. The anonymity was ensured this way: at the beginning of the demographics section, the participants were asked to create their nicknames following the pattern given by the researcher.

The second measurement was only taken among persons who were finishing the basic treatment. The procedure was the same as in the first measurement.

## Results

The means and standard deviations of the studied variables divided by sex and measurement are presented in Table 2.

**Table 2:** Descriptive statistics and the comparison of therapy effects for men and women

Variables	Scale range	Men			Cohen’s *d*	Baseline *M* (SD)	Women		Cohen’s *d*
		Baseline *M* (SD)	Posttest *M* (SD)	*t*			Posttest *M* (SD)	*t*	
POS. spiritual coping	1–5	3.45 (.83)	3.67 (.69)	2.58*	.34	3.54 (.66)	3.94 (.63)	5.03***	.67
NEG. spiritual coping	1–*5*	2.15 (.85)	1.87 (.63)	−2.41*	−.32	2.51 (.82)	2.03 (.75)	−4.51***	−.60
Positive personal	1–5	3.81 (.86)	3.97 (.77)	1.22	.16	3.86 (.72)	4.29 (.58)	5.48***	.71
Positive social	1–5	3.96 (.86)	3.91 (.66)	−.47	−.06	3.92 (.64)	4.15 (.71)	2.61*	.27
Positive environmental	1–5	3.15 (1.21)	3.46 (1.09)	2.11*	.28	3.17 (1.04)	3.60 (.95)	3.90***	.52
Positive religious	1–5	2.88 (1.40)	3.23 (1.31)	2.43*	.32	3.20 (1.34)	3.83 (1.07)	4.30***	.57
Negative personal	1–5	2.41 (1.30)	2.11 (.99)	−1.75^a^	−.23	2.95 (1.15)	2.17 (.89)	−5.06***	−.68
Negative social	1–5	1.93 (.89)	1.82 (.81)	−.89	−.12	2.24 (.90)	1.96 (.92)	−2.04*	−.27
Negative religious	1–5	1.87 (1.01)	1.69 (.75)	−1.21	−.16	2.34 (1.21)	1.95 (1.04)	−2.61*	−.34
Forgiveness	1–5	3.12 (.64)	3.13 (.66)	.22	.03	2.98 (.70)	3.21 (.68)	2.66*	.36
Forgiveness of self	1–5	2.44 (1.00)	2.47 (1.18)	.22	.03	2.30 (1.07)	2.26 (1.02)	−.28	−.04
Forgiveness of others	1–5	3.43 (.81)	3.40 (.87)	−.27	−.04	3.13 (.71)	3.50 (.74)	4.12***	.55
Feeling of being forgiven by God	1–5	3.53 (1.24)	3.46 (1.18)	−.48	−.06	3.46 (1.39)	3.92 (1.22)	2.84**	.38
Gratitude	6–42	30.19 (7.05)	31.02 (6.35)	1.17	.16	29.39 (6.66)	31.64 (6.50)	3.08**	.41

^a^ *p* ∈ [.05; .1); * p < .05; ** p < .01; *** *p* < .001

The initial stage of calculations involved comparing the intensity of domains of spiritual coping and aspects of forgiveness. For this purpose, comparative analyses with the use of Student’s *t* test and ANOVA with post hoc tests (the Games–Howell test) were conducted. A higher level of positive spiritual coping than negative one was observed for men and women, both at baseline (men: *t* (110) = 8.162; *p* < .001; women: *t* (110) = 7.265; *p* < .001) and in posttest (men: *t* (110) = 14.302; *p* < .001; women: *t* (110) = 14.597; *p* < .001).

In measurement I, in the group of women and men alike, social and personal domains of positive spiritual coping had the highest levels (see Table 2). In both groups, the level of these domains of spiritual coping was significantly higher than the level of environmental (men: *p* < .001; women: *p* < .01) and religious one (men: *p* < .001; women: *p* < .01). In measurement II, both men and women declared they most often applied personal and social positive spiritual coping (Table 2).

The highest level of negative spiritual coping in women and men alike was observed for the personal domain in both measurements (Table 2). At baseline, the level of negative personal spiritual coping was significantly higher than negative spiritual coping in both groups as regards the social domain (men: *p* = .064; women: *p* < .01) and the religious one (men: *p* < .05; women: *p* < .05). In addition, among men, it was higher than negative religious spiritual coping in posttest (*p* < .05).

Both in women and in men, the lowest value among all the aspects of forgiveness was found for self-forgiveness (Table 2). In both sexes, the level of this variable proved to be lower (*p* < .001) than the level of forgiving others and the feeling of being forgiven by God.

### Comparison of Sex Differences in Spiritual Coping, Forgiveness, and Gratitude in Measurements I and II

**Fig. 1:**
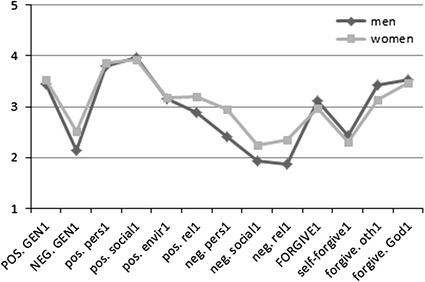
Comparison of men’s and women’s spiritual coping, forgiveness, and gratitude in the first measurement. *POS*. *GEN* positive spiritual coping, including its particular domains: *pos*. *pers* personal, *pos*. *social* social, *pos*. *envir* environmental, *rel* religious, *NEG*. *GEN* negative spiritual coping, including following domains: *neg*. *pers* personal, *neg*. *social* social, *neg*. *rel* religious, *FORGIVE* forgiveness with its aspects: *self*-*forgive* self-forgiveness, *forgive*. *oth* forgiveness of others, *forgive*. *God* the feeling of being forgiven by God

**Fig. 2:**
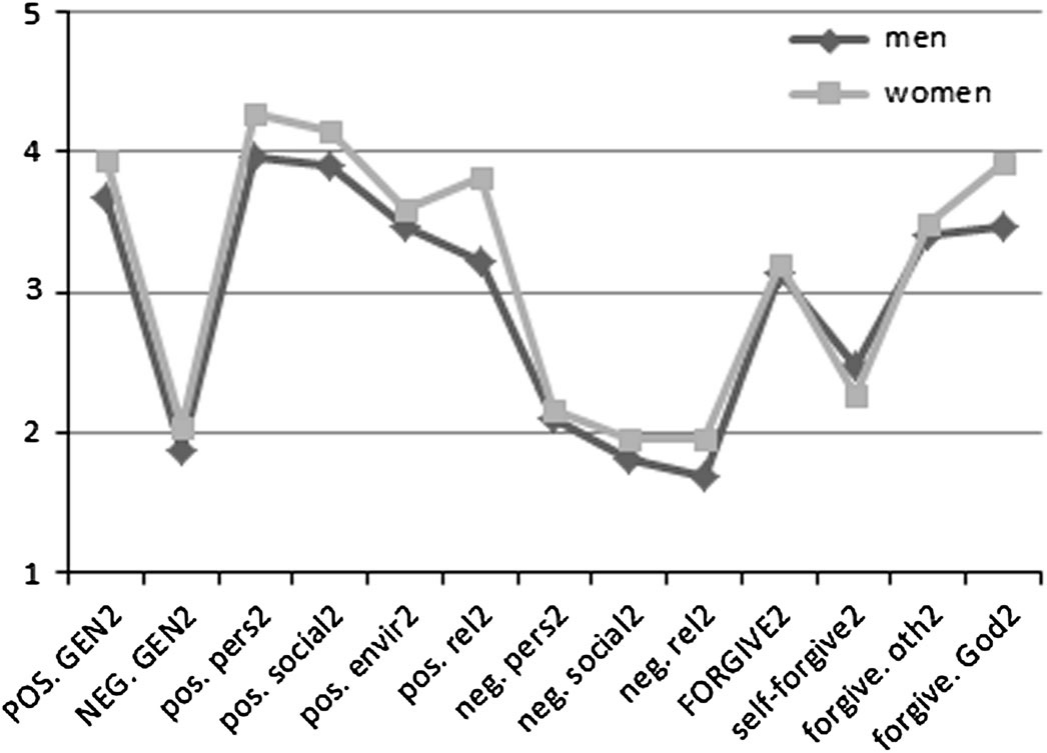
Comparison of men’s and women’s spiritual coping, forgiveness, and gratitude in the second measurement. Explanation of abbreviations—see Fig. 1

The levels of particular domains of spiritual coping, forgiveness, and gratitude in both measurements for women and men were compared with the use of Student’s *t* test for two independent samples. The findings are presented in Figs. 1 and 2.^1^

When entering the basic therapy, women had a higher general level of negative spiritual coping than men (*t* (110) = −2.281; *p* = .024), including negative spiritual coping in the personal (*t* (110) = −2.309; *p* = .023), religious (*t* (110) = −2.248; *p* = .027), and social domains (*t* (110) = −1.839; *p* = .069). No significant differences in positive spiritual coping were found between men and women. However, a significant difference in forgiving others was noted: men were characterized by a higher level of this variable than women (*t* (110) = 2.068; *p* = .041).

At the end of the therapy, however, sex differences changed. Compared to men, women had a higher general level of positive spiritual coping (*t* (110) = −2.211; *p* = .029), including its religious (*t* (105.501) = −2.677; *p* = .009), personal (*t* (110) = −2.479; *p* = .015), and social domains (*t* (110) = −1.184; *p* = .068). No significant sex differences in negative spiritual coping and gratitude were found. However, a significant difference between women and men was noted in the feeling of being forgiven by God: the former group demonstrated a higher intensity of the variable than the latter one (*t* (110) = −2.031; *p* = .045).

### Changes in the Studied Variables During the Therapy

Using the Student’s *t* test for two dependent samples, the therapy effectiveness regarding the studied variables was calculated for women and for men. The obtained results (*see* Table 2) showed that positive changes in spiritual coping were more intensive in women than in men. In that group, the value of Cohen’s *d* (1988) ranged from .27 for the social domain up to .71 for the personal domain of positive spiritual coping. In the case of forgiveness, the level of this statistics was from .38 for the feeling of being forgiven by God to .55 for forgiving others, whereas for gratitude it was .41. These results indicate a moderate effect size (Cohen 1988). In the case of men, positive changes only occurred in the general level of positive spiritual coping with stress, including its environmental and religious domains, and the general level of negative spiritual coping, including its personal domain on the statistical tendency level. The strength of effect calculated for the significant variables in that group proved to be lower than in the case of women: it ranged from −.23 for negative spiritual coping in the personal domain up to .34 for the general level of positive spiritual coping. No significant changes in the studied moral values were observed in this group.

## Discussion

The preliminary analysis of the results showed a higher level of positive spiritual coping than the negative one in both measurements in women and men alike. These results are supported by previous studies (Zwingmann et al. 2006). It seems, then, that even in alcoholism, which often destroys one’s spiritual sphere, people more often use positive sources of spirituality than its negative expressions.

In this context, it is interesting to compare the intensity of particular domains of spiritual coping. In both measurements, the personal domain of positive spiritual coping had a relatively high level. The highest values of negative spiritual coping were also found for the personal domain. The results are similar for women and men. It means that people entering alcohol addiction therapy in an effort to cope with the stress connected with the disease try to find the meaning of their lives, seek their place in the world, try to learn and understand themselves better and achieve internal harmony (positive personal coping), and at the same time, they experience the sense of meaninglessness of their existence, leading to thoughts about their worthlessness and the will of self-destruction (negative personal spiritual coping). Two forces can be seen here: the “centripetal” one, causing people addicted to alcohol to look for guidance and strength in spiritual resources, and the “centrifugal” one, leading to negation of the purpose of one’s life.

It is also noteworthy that in both measurements, the addicted people had a rather low level of religious spiritual coping, both in the positive and negative aspects. This may mean that at least during the basic treatment program, addicted people are more concentrated on the personal and social aspects of spirituality, and the religious domain plays a smaller role then. The religious domain may become important for the processes of coping at further stages of treatment. This hypothesis needs to be further explored.

What is notable in the analysis of the level of aspects of forgiveness and changes occurring during the treatment is the lowest level of self-forgiveness and the lack of changes in this variable, both in the group of women and of men. This finding is consistent with the results of previous studies (Webb et al. 2006). Forgiving oneself can be the aspect of forgiveness that is most difficult to change and at the same time plays an important role in the process of treatment (Webb et al. 2009). The obtained results indicate the need for greater concentration on this issue during the treatment, with clear identification of the difference between mature self-forgiveness as a conscious process and its unfavorable form connected with defensive mechanisms, which may contribute to continuing detrimental behaviors by means of defense and rationalization (McNulty 2011).

Women starting the alcohol addiction therapy had a higher general level of negative spiritual coping (as well as a higher level of all its subscales) and a lower level of forgiveness of others when compared to men. Interpreting the obtained results, it should be remembered that women generally demonstrate a higher level of religiousness and spirituality, and they more often than men refer to the spiritual sphere in stressful situations (Strawbridge et al. 2000; Krause 2006; Koenig et al. 1999). The fact that women rely more on spiritual resources in stressful situations may not be true for women addicted to alcohol, at least at the moment of entering therapy.

Some studies have shown that in women, one of the most important factors promoting the development of addiction and causing relapses is problems in interpersonal relations (Covington and Surrey 1997; McKay et al. 1996). Thus, negative feelings toward other people, including family members and friends, can lead this group to accumulating resentment and negative spiritual coping in the social domain. In the face of deepening disappointment with close relationships, addicted women may turn away from spirituality as a positive source of energy for life. They treat people as egoistic, they feel deserted and abused. It can also be related to their life experiences, among others with addicted women more frequently experiencing physical and mental violence and sexual abuse in comparison with addicted men (Wechsberg et al. 1998).

Furthermore, women addicted to alcohol may blame themselves for failing to meet social expectations connected with the roles of a wife or mother, and they may feel they did not meet their own standards. They lose the sense of meaningfulness and purposefulness of their lives. In addition, although these women may have had relatively good, stable relations with God in the past, the development of addiction may have caused the emergence of anger with God and increasing sense of loneliness, often resulting in questioning His love and care.

Despite the fact that the above hypothesis (saying that the development of addiction affects the emergence of negative methods of spiritual coping and difficulties with forgiving others especially in women) seems plausible, the reverse direction cannot be excluded, assuming that women with a higher level of negative spiritual coping than the population average more often get addicted. Longitudinal studies are necessary to verify this hypothesis.

In this study, positive changes in spiritual coping were observed during the therapy. These results are reinforced by a few previous studies concerning spirituality (Robinson et al. 2011a; Sterling et al. 2007). Although positive changes in the general level of spiritual coping were observed both for women and for men, in the case of women the improvement occurred in all the domains of spiritual coping, whereas in men, it only referred to the positive environmental and religious domain, and to a lesser degree, the negative personal one. Changes found in women were stronger in all the domains of spiritual coping. The greatest changes in this group were in the personal and religious domains, which means that during the therapy, women were learning how to concentrate on their internal lives, to look for harmony and peace, to reduce the sense of purposelessness of their lives, and at the same time to draw closer to God/Higher Being. Men first of all learnt to use their spiritual resources connected with the development of close relations with Nature and the Higher Being to a greater extent than before.

As for changes in forgiveness and gratitude during the therapy, considerable differences between the sexes were noted. Although women began the treatment with a lower level of forgiving others than men and an equal level of the other variables, during the therapy positive changes in moral values (except for self-forgiveness) only occurred in the group of women.

The obtained results concerning forgiveness and gratitude are consistent with those regarding spiritual coping. They lead to the conclusion that although in the group of women some elements of spirituality are more susceptible to the destructive influence of alcohol, it is easier to restore their importance during the therapy. It seems that the discussed sex differences in the dynamics of changes are among others the result of the greater role attributed by women to religiousness and spirituality. The results suggest that this sphere can therefore be more prone to changes in women, which may have both negative consequences for the individual (e.g., in the case of pathological drinking) and positive ones (e.g., in the case of treatment). For most men, the spiritual sphere is not as important in everyday life as for women (Beit-Hallahmi and Argyle 1997), which may mean on the one hand that in this group, it is less susceptible to the destructive influence of alcohol, but on the other hand, it is more resistant to the therapeutic influence of the treatment.

When trying to explain the mechanism of changes occurring in women during the treatment, we can refer to the experience of spiritual awakening. According to Alcoholics Anonymous (1972), it does not have to occur rapidly, but it may be treated as a gradual process of spiritual transformation, which involves a change in the self, especially in terms of relations with others and with God/Higher Being (Neff and MacMaster 2005). The damaged interpersonal relations may be repaired thanks to social support and peer influence processes occurring during the therapy and AA meetings (Bond et al. 2003). Women have an opportunity to improve their relations with others, establish acquaintances and friendships, restore the faith and trust in other people and in themselves as persons able to provide support to others, and to apply moral values in life. This helps them to stop perceiving themselves as valueless, morally deviant individuals full of grudges and blaming the world and God for their failures. Looking for strength rooted in spiritual sources may lead to improving the self-esteem and self-acceptance, and may help to find the meaning and purposefulness of one’s life. It also promotes the realization that regaining the positive image of self, others, the world, and God/Higher Being by means of hard work on ones’ emotions and the development of constructive methods of coping with stress (including those that are based on spiritual resources) can really be achieved, which may contribute to the development of a more internal orientation regarding the drinking-related locus of control (Koski-Jännes 1994) and strengthening the belief in abstinence self-efficacy (Piderman et al. 2008). These processes may foster further spiritual development and maintaining sobriety (Linquist 2013; Strobbe et al. 2013). The presented proposal for the process of spiritual change occurring in women during the therapy is a hypothesis, which needs to be verified in further studies.

## Conclusions

There can be no doubt that more emphasis on the issues connected with spirituality might bring positive results for the treatment. It is so because spirituality may be a source of strategies of coping with stress which are significant from the point of view of maintaining abstinence and the process of sobering (Robinson et al. 2011b; Wnuk 2007). In light of the obtained results, it seems that currently the implementation of spiritual issues in therapy would be especially beneficial for women. This group has a greater sense of stress and shame when starting the therapy; besides, women addicted to alcohol report more problems and difficult situations than men (Thom 1987). The development of appropriate coping strategies, including strategies based on spirituality, would create the opportunity for better confrontation with a stressful situation of addiction. These assumptions were preliminarily confirmed in the study that showed negative relationships between spirituality and perceived stress in women undergoing alcohol addiction therapy (Arévalo et al. 2008).

On the basis of the presented study, it can be also concluded that during the therapy, men develop their spiritual potential to a lesser degree than women do. According to the results of other studies, men are less inclined to forgive and more often hold a grudge against the person who has harmed them (Miller et al. 2008), and also feel and express gratitude to a lesser degree than women do (Kashdan et al. 2009). This may make the growth of spiritual functioning during the therapy more difficult for them. In the case of this group, work of therapists and patients themselves on increasing the internal peace and harmony, the sense of meaning and purpose of one’s life, deepening relationships with others based on moral values, and tightening the relationship with the force higher than the self would be especially beneficial.

Interpreting the results of the presented study, one must remember about its limitations. The studied sample was rather small and only involved people who were completing treatment at open units. It would be recommendable to replicate the study with a greater sample, with consideration of different groups: people undergoing treatment at inpatient units, people maintaining abstinence for many years, people only attending AA meetings, and people who do not use any form of treatment.

Another limitation of the study was the effect strength: it did not exceed .8 (which is regarded as “grossly perceptible and therefore large” effect; Cohen 1988, p. 27) for any of the variables. Sex differences at the beginning and at the end of the treatment were not huge either, ranging from the statistical tendency level for positive spiritual coping in the social domain in both measurements to the level of *p* = .009 in the case of positive spiritual coping in the religious domain at posttest. It should also be remembered that the study was carried out at different centers, which—despite using a similar basic treatment program—differed in the duration of the treatment (from 6 to 8 weeks). It is possible that at least some changes that occurred in people who took part in an 8-week program did not yet occur (or were weaker) among the patients who were undergoing a 6- or 7-week program.

Another limitation of the study is that only two measurements were taken, at the beginning of the basic treatment program and after its completion. Conducting further measurements would enable researchers to perceive changes occurring after the completion of the basic treatment program.

When interpreting the results of the study, the cultural context should also be taken into account. The study was conducted in Poland, a country where more than 90 % of the population declare to be Catholics (CBOS 2012). In this group, referring to spiritual resources, including the application of moral values in a stressful situation of taking up alcohol addiction treatment, may be quicker and stronger than in groups with lower faith levels. This hypothesis is supported among others by a study conducted in a group of Poles beginning addiction therapy, which showed that this group may be especially prone to experience spiritual awakening during treatment (Strobbe et al. 2013).

In further work on spiritual functioning of people addicted to alcohol, it would be advisable to study interindividual differences in intra-individual patterns of change across time. Advanced statistical methods (e.g., Latent Growth Curve Modelling; Meredith and Tisak 1990) would be helpful in this respect.

Finally, it is necessary to carry out studies to show how the changes in different aspects of spiritual life of the addicted people affect maintaining abstinence and the recovery process, as well as multidimensional functioning, especially for the variables whose significance for the treatment process is not recognized yet, i.e., spiritual coping and gratitude.

## Footnotes

1 The variable “gratitude” was not presented in the figures, because it was expressed on a different scale than the other variables.
